# Transmembrane 163 (TMEM163) Protein: A New Member of the Zinc Efflux Transporter Family

**DOI:** 10.3390/biomedicines9020220

**Published:** 2021-02-21

**Authors:** Daniel J. Styrpejko, Math P. Cuajungco

**Affiliations:** 1 Department of Biological Science, California State University Fullerton, Fullerton, CA 92831, USA; dannystyrpejko@csu.fullerton.edu; 2 Center for Applied Biotechnology Studies, California State University Fullerton, Fullerton, CA 92831, USA

**Keywords:** zinc, cation diffusion facilitator, zinc transporter, SV31

## Abstract

A growing body of evidence continues to demonstrate the vital roles that zinc and its transporters play on human health. The mammalian solute carrier 30 (SLC30) family, with ten current members, controls zinc efflux transport in cells. TMEM163, a recently reported zinc transporter, has similar characteristics in both predicted transmembrane domain structure and function to the cation diffusion facilitator (CDF) protein superfamily. This review discusses past and present data indicating that TMEM163 is a zinc binding protein that transports zinc in cells. We provide a brief background on TMEM163’s discovery, transport feature, protein interactome, and similarities, as well as differences, with known SLC30 (ZnT) protein family. We also examine recent reports that implicate TMEM163 directly or indirectly in various human diseases such as Parkinson’s disease, Mucolipidosis type IV and diabetes. Overall, the role of TMEM163 protein in zinc metabolism is beginning to be realized, and based on current evidence, we propose that it is likely a new CDF member belonging to mammalian SLC30 (ZnT) zinc efflux transporter proteins.

## 1. Introduction

Zinc is a micronutrient involved in many fundamental roles that are vital for routine bodily functions. It is estimated that one in ten proteins have zinc-binding motif [1]. Additionally, every one of the main six classes of enzymes (hydrolases, isomerases, ligases, lyases, oxidoreductases, and transferases) contains zinc-dependent proteins [2]. At the molecular level, zinc binds to proteins [1,3] in various capacities such as protein structural integrity, catalysis, and regulatory activity of DNA function [2,4]. Additionally, zinc plays other crucial roles in cell signaling pathways such as the “zinc spark” during fertilization [5], modulation of neurotransmitter receptors or ion channels [6,7,8] and second messenger system of specific signal transduction pathways [1]. The widespread effects of and dependence on zinc by various proteins demonstrate the broad usefulness of this trace metal. We refer the reader to a review on the multi-dimensional effects of zinc in cells by Cuajungco, Ramirez, and Tolmasky (2021) as part of the Special Issue.

Mammals, especially in humans, require zinc for proper immune responses. Innate and adaptive immune systems both rely on proper zinc levels in order to develop and carry out their protective duties [2,3]. Evidence also demonstrates that zinc can benefit those suffering from viral, bacterial, and even parasitic infections [2]. In addition to helping to destroy endocytosed pathogens, zinc can be used in ridding cells of reactive oxygen species (ROS) [2]. However, for some cell types, there is indication demonstrating that intracellular zinc accumulation leads to increased ROS formation in the mitochondria [9,10]. The strict control of ROS production demonstrates another crucial, although indirect role for zinc, especially that ROS imbalance releases zinc from metalloproteins and creates oxidative stress within the cells leading to disease [11,12]. 

Zinc dyshomeostasis is a known factor in many human health problems. In diabetes mellitus (DM), there is evidence associating zinc imbalance occurring at the physiological and cellular levels [11,13]. For example, insulin needs two zinc ions to stabilize its granules, but the role of zinc in DM is more complicated than simply zinc dyshomeostasis or abnormal metabolism [14], which will be discussed later in this review. Zinc deficiency also has consequences on the immune system. One such example is that zinc deficiency adversely affects the function of immune cells as evidenced by atypical inflammatory responses occurring under reduced zinc conditions [15]. Additionally, zinc deficiency has been shown to be associated with chronic diseases such as liver cirrhosis [16] and asthma [3]. While cases of zinc deficiency in human population are often more mild-to-moderate in their severity, it is relatively widespread and affects one in four people [3,11]. With zinc deficiency afflicting many people worldwide and a wide variety of health issues stemming from it, nutritional supplementation appears to be quite important. Nevertheless, zinc supplementation alone is not enough to solve the many issues in human health [3], because abnormal zinc levels in the human body could also mean increased tissue or cellular zinc concentrations that result in cytotoxicity. Thus, it is necessary to further understand zinc homeostasis with respect to the living cell. 

## 2. Zinc Transport

Further insight into human diseases linked with zinc imbalances can be obtained by studying how zinc is transported into and out of cells. The solute carrier 30 (SLC30) and the SLC39 families comprise the two major groups of cellular zinc transporters [17]. As such, these families play vital roles in regulating tissue zinc homeostasis. Due to their considerable control of zinc concentrations, it is not surprising that a large number of diseases can result from, or are associated with, members of these two families that are either dysfunctional or differentially expressed [18,19]. Due to the similarities of human Transmembrane 163 protein (TMEM163) to SLC30 proteins, as will be discussed later on, only the SLC30 family will be further explored in this section.

### 2.1. The SLC30 Family or ZnT Efflux Transporters

The SLC30 proteins or ZnTs belong to the CDF superfamily [18,20]. These are zinc transporters found across a wide variety of species from bacteria to humans. ZnTs have six transmembrane domains (TMDs) with intracellular amino terminus domain (NTD) and relatively longer carboxyl terminus domain (CTD) [18,21]. Although a longer CTD is expected of CDF proteins, there have been protein homologs identified in bacteria (marine and soil) that lack longer CTD [22]. There are currently ten known ZnT proteins [18,20] with ZnT1–ZnT4 identified through direct experimentation with zinc-resistant cells or via cloning, while ZnT5–ZnT10 were found by homology sequence analysis using previously discovered ZnTs [18]. The structure of a bacterial CDF called YiiP has been published [23], which served as a template to model theoretical structures of certain mammalian ZnT proteins. Recently, however, the structure of human ZnT8 was also solved using cryo-electron microscopy [24]. The recent publication of the ZnT8 structure confirmed that certain members of the CDF family, especially the SLC30 proteins, have specific zinc-binding sites within their TMDs and the CTD [24].

As a whole, the ZnTs function almost exclusively as zinc effluxers, and as such, they export zinc from the cytosol to either the outside of the cell or bring cytoplasmic zinc into vesicles or organelles [18,20]. Note, however, that ZnT10 is unusual from its related members in that it has been reported to efflux not only zinc [25], but also manganese ions [17,26,27]. Recent data suggest that ZnT10 mainly extrudes manganese [27] and that manganese efflux appears to be a calcium-dependent antiport process [28] as opposed to a proton-dependent mechanism previously shown for certain ZnT proteins [29,30,31]. The functional difference between ZnT10 and other ZnTs may stem from the putative metal binding motif on ZnT10′s TMD2, which is NXXXD compared to the typical HXXXD associated with zinc binding observed for specific ZnTs [17]. Counter to expectation, site-directed mutagenesis (SDM) targeting both asparagine (N) and aspartate (D) of the TMD2 NXXXD motif within ZnT10 did not inactivate its manganese efflux function [26,27]. These results indicate that the TMD2 HXXXD motif that has been ascribed to zinc binding is not generalizable and cannot be used solely to classify a protein as a ZnT family member without sufficient empirical evidence to support classification. A case in point, ZnT6 does not have the TMD2 HXXXD motif, but rather, it has a TMD2 DXXXD motif. As we will elaborate later on, the TMD2 DXXXD motif is also found in both YiiP and TMEM163 [32]. Despite such debatable findings, the role of ZnT10 as an effluxer is not in doubt; however, it may be that this protein is an atypical ZnT member as is the case for ZnT9 and TMEM163 as discussed below and in the subsequent section. Future research should investigate specific motifs within TMD2 and TMD5, as well as relevant amino acid residues surrounding these domains to fully define their role in zinc transport and help establish a way to define classification and membership to the ZnT family or the CDF family, in general. 

A truncated isoform of ZnT5 [33] has been reported to act as a zinc influxer, while a variant of ZnT8 [34] implicated in DM appears to show a similar influx activity when heterologously expressed in cells. While such bidirectional activity may suggest a novel function for some of these members of the ZnT family, this characteristic has been called into question upon further examination of the structure of ZnT8 [24,35]. Thus, members of the SLC30 family should be recognized as mainly effluxers until more extensive evidence showing bidirectional function is experimentally validated. Of added interest is that ZnT9 had its membership with the SLC30 family called into question due to its reported role as a nuclear receptor co-activator and possible lack of zinc transport function [17]. Recently, however, research into the causal role of a point mutation within ZnT9 leading to a cerebral-renal disease in humans has shed some light in support of ZnT9 as a zinc transporter [36]. Moreover, knock-down of ZnT9 expression in human chondrocytes results in upregulation of the zinc-regulatory transcription factor, MTF-1, while over-expression of ZnT9 produces an opposite effect, suggesting that ZnT9 mediates intracellular zinc flux [37]. Noteworthy is that ZnT9 also has the HXXXD zinc-binding motif on TMD2 that is found among members of the ZnT family [17]. To resolve this controversy, further studies would need to be done to validate these observations and to show specific domains or motifs that are directly responsible for the zinc efflux function of ZnT9.

ZnTs localize to different areas in the cell. Some common subcellular localization of ZnTs include the plasma membrane, synaptic or secretory vesicles, and the Golgi apparatus [17,18,20]. A central structural feature of ZnTs is that they transport zinc as a dimer. Noteworthy is that certain ZnT subunits heterodimerize with each other to carry out their functions, although some ZnT subunits can only form functional homodimers while others could form more than one heterodimer version [18,38,39]. Strong evidence shows that ZnT1–ZnT4, ZnT7 [39], and ZnT8 [40] form homodimers, but that ZnT1–ZnT4 are also able to form heterodimers with each other [38]. Similarly, ZnT5 may form a homodimer but it also heterodimerizes with ZnT6 [39,41]. ZnT3 and ZnT10 are reported to form heterodimers with each other [42]. As heterodimers, some ZnTs confine themselves differently from their normal subcellular localization [38], exhibiting features that are far more intricate than merely effluxing zinc. However, the exact nature of these ZnT subunit interactions and the express purposes of creating functionally redundant ZnT heterodimers in various cell types remain to be elucidated. Thus, further investigation into ZnT dimerization, differential subcellular localizations, and the consequences of subunit interactions should be priorities in future studies of the ZnT protein family. 

### 2.2. ZnTs in Human Diseases

**Figure 1:**
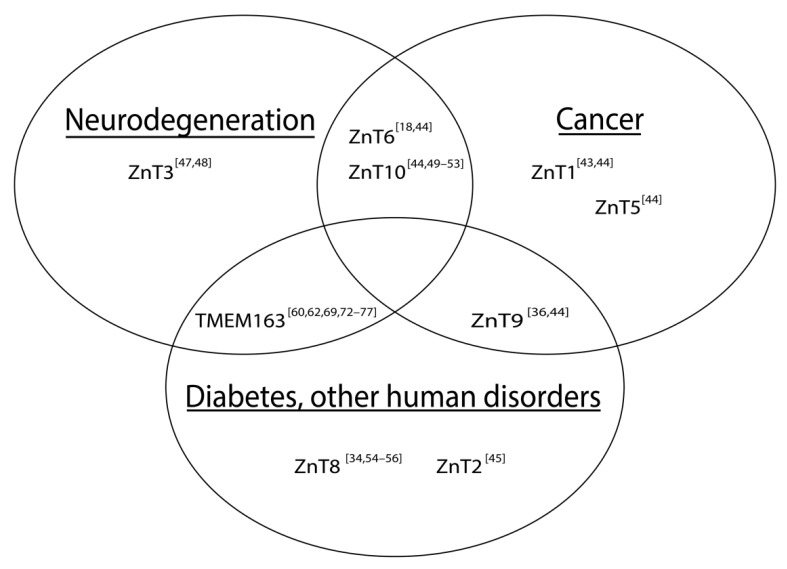
Schematic illustration of associations between altered SLC30 (ZnT) expression and notable human diseases. The Venn diagram includes TMEM163, which has been implicated in certain human diseases. References are represented as numbers in brackets.

Many diseases and health issues have been linked with the improper function or altered expression of ZnTs (Figure 1). More specifically, various ZnTs have been implicated in neurodegenerative diseases and cancers. Both a loss of function [43] and an over-expression of ZnT1 [44] for example, are associated with cancer, but that the former findings may be predictive or prognostic indicator of patient survivability [43]. Mutations in ZnT2 cause zinc concentrations in breast milk to be deficient, which leads to zinc deficiency in children if their primary source of the micronutrient comes from breast milk [45]. Expression levels of ZnT3 are shown to be reduced in post-mortem brains of Alzheimer’s disease (AD) patients [46] and in a mouse model of Mucolipidosis type IV (MLIV) disease [47]. On the other hand, ZnT3 levels appear to be elevated in the cerebellum of a mouse model of AD [48]. Meanwhile, increased expression levels of ZnT6 [18] and decreased expression of ZnT10 [49] have been associated with AD pathology in the hippocampus and frontal cortex, respectively. It is also worth noting that specific mutations within ZnT10 are linked with Parkinson’s disease (PD), dystonia with or without hyper-manganesemia, chronic liver disease, and polycythemia [50,51,52,53]. In prostate cancer, ZnT5 and ZnT6 expression levels are downregulated, while ZnT9 and ZnT10 expression levels are shown to be increased [44]. As mentioned earlier, a mutation in ZnT9 was recently found to be responsible for cerebro-renal syndrome disease [36] and its function may modulate the expression of aggrecan, which is implicated in Temporomandibular joint osteoarthritis [37]. ZnT8 has been widely reported to be involved in DM [34,54,55,56]; however, its role in type II diabetes (T2D), particularly linked with a loss-of-function ZnT8 variant [55], conflicts with evidence supporting treatment of T2D by increasing ZnT8 function [54].

The list of zinc transporters that appears to be correlated with various human diseases in the current review is not exhaustive. Nevertheless, it demonstrates that zinc plays many roles in influencing normal and diseased states, and thus, broadening the investigation of these proteins is of significant value to devise or discover a form of therapeutics against many debilitating diseases in humans. 

## 3. TMEM163

### 3.1. Protein Characteristics and Function

Human TMEM163 protein, recently characterized as a zinc efflux transporter [32], is also known as synaptic vesicle 31 (SV31) [57]. Its rodent counterpart, Tmem163, was first discovered in rat brain synaptosomes, which explains its initial coining as Sv31 protein [57]. Rodent Tmem163 is detected in certain neuronal populations that express glutamatergic and γ-aminobutyric acid receptors [57,58], where zinc is also present [59]. Barth and colleagues (2011) used immunocytochemistry and subcellular fractionation studies of neuron-like PC-12 cells stably expressing rodent Tmem163 to reveal that this protein is detected mostly in the plasma membrane (PM), lysosomes, early endosomes, and other vesicular compartments [58]. Confocal microscopy studies of heterologously expressed TMEM163 revealed PM and lysosomal localization [60]. The *TMEM163* gene has high relative transcript expression in the lungs, followed by the brain (cortex and cerebellum), and then the testis [60]. However, the reported transcript expression patterns of *TMEM163* in human tissues appear to vary depending on the housekeeping gene used to quantify relative mRNA levels—our laboratory used *18S rRNA* while another group used *GAPDH* [61]. Nevertheless, the tissue expression of TMEM163 protein corresponds with those of other ZnT proteins [62]. Note also that the relative mRNA tissue expression of mouse *Tmem163* gene parallels that of its human counterpart [32], despite the initial claims that the gene is only exclusively expressed in the mouse brain [57,58].

TMEM163 is a 31.5 kDa protein that binds divalent cations such as zinc, nickel, and copper, although the protein does not bind copper as strong as zinc or nickel [58]. It has a predicted six TMDs with intracellular amino and carboxyl termini, and forms a functional homodimer [63]. In silico analysis of protein sequences showed that Tmem163 and other members of the CDF family share homologous amino acid sequence, while alignment of the TMDs of YiiP, CzcD, ZnT3, and Tmem163 suggests that they have similar predicted topological structure [58]. However, unlike members of the CDF family, both TMEM163 and its rodent Tmem163 counterpart lack some features common to the ZnT protein family. Namely, TMEM163 has a longer predicted NTD and shorter CTD, albeit this topology does not necessarily exclude it from being a member of the CDF family because as mentioned earlier, numerous non-mammalian CDF proteins with short or atypical CTD have been discovered [22]. TMEM163 also does not have a histidine-rich amino acid residues that appear to be common among ZnT proteins. However, TMEM163 does have several histidine residues proposed to mediate zinc binding and transport as evidenced by histidine-specific chemical modification experiments reported by Waberer and colleagues (2017) [63]. As mentioned earlier, majority of the ZnT members has the HXXXD motif within their TMD2 and TMD5 [17]. Multiple sequence alignment (MSA) of protein sequences from all ten ZnT family members, TMEM163, and several non-mammalian CDF family members (YiiP, CzcD, and ZitB) revealed that TMEM163, ZnT6, and YiiP have the DXXXD motif on their predicted TMD2 instead of the HXXXD typically found within TMD2 and TMD5 of ZnT and certain CDF proteins [32]. Notwithstanding, the ability of TMEM163 to transport zinc has been shown in both rodent [63] and human [32] species through a zinc flux assay using different kinds of fluorescent dyes [32,58,60,63,64]. In particular, alanine substitution using SDM of the TMD2 DXXXD motif of both rodent Tmem163 and human TMEM163 proteins showed that the aspartic acid residues are critical for zinc binding [32,63].

One aspect of controversy surrounding the zinc transport function of TMEM163 is whether it acts as an influx or efflux transporter. For example, initial reports using PC12 cells transiently or stably expressing rodent Tmem163 produce intracellular zinc accumulation when these cells were exposed to exogenous zinc [57,58]. This result suggests that Tmem163 is a zinc influx transporter. Indeed, our laboratory previously observed HEK-293 cells transiently expressing TMEM163 exhibit an increase of intracellular zinc upon zinc exposure during live cell imaging, leading us to primarily think that it might be an influx transporter [62]. Despite these observations, more confirmation is needed if TMEM163 is a bona fide zinc influxer. A recent report of zinc transport by rodent Tmem163 was done using heterologously expressed purified protein that was assayed within artificial lipid nanodiscs (liposomes) [63]. Exogenous zinc exposure of purified Tmem163 localized within liposomes produced intra-luminal zinc accumulation as evidenced by a zinc fluorophore, suggesting that the protein facilitates ionic influx [63]. While the study was an elegant approach and the direction of the Tmem163-mediated transport was indicative of influx, the model system used was not a living cell comprising of PM, intracellular organelles or compartments, and endogenous proteins. More recently, however, cultured human cell lines transiently or stably expressing TMEM163 that were exposed to exogenous zinc revealed that TMEM163 serves to extrude zinc out of the cells [32]. Radioactive zinc-65 flux assay of cultured cells transiently expressing TMEM163 supports the report that TMEM163 is a zinc efflux transporter (Saima Ali and Math P. Cuajungco, unpublished data).

**Figure 2:**
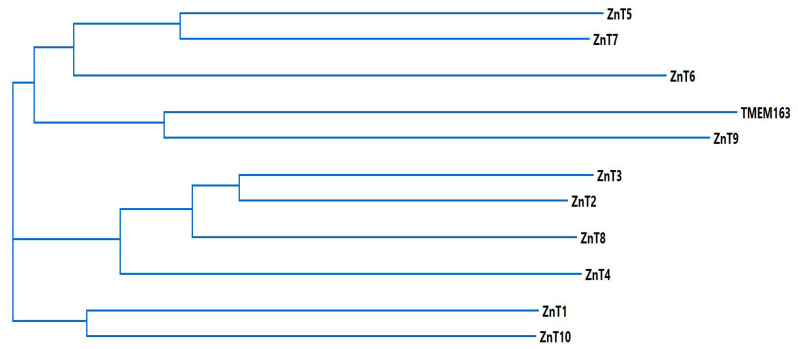
Cladogram of TMEM163 and ZnT family members. The multiple sequence alignment and phylogenetic tree were calculated using Clustal Omega (Lasergene v17.1 MegAlign Pro; DNAStar, Madison, WI, USA). Full-length amino acid sequences were obtained from the UniProt website: TMEM163 (Q8TC26); ZnT1 (Q9Y6M5); ZnT2, (Q9BRI3); ZnT3 (Q99726); ZnT4 (O14863); ZnT5 (Q8TAD4); ZnT6 (Q6NXT4); ZnT7 (Q8NEW0); ZnT8 (Q8IWU4); ZnT9 (Q6PML9); and ZnT10 (Q6XR72).

With respect to the relationship of TMEM163 with the CDF family, especially with the ZnT proteins, MSA and phylogenetic analyses demonstrated that TMEM163 may be evolutionary related to ZnT9 [32]. As a means to further explore the association, we performed MSA and phylogenetic analyses using MegAlign Pro (Lasergene v17.1) to compare TMEM163 with ZnT proteins. The cladogram of the phylogenetic tree (Figure 2) suggests that TMEM163 and ZnT9 are associated and that both proteins diverged from a common ancestral protein that also gave rise to ZnT6 as well as related proteins, ZnT5 and ZnT7. 

A tabulated matrix showing the percentage of identity and similarity of the amino acid sequences of TMEM163 and ZnT proteins are shown on Table 1. Similarity and identity scores were typically higher among members of the ZnT protein family. The matrix confirms that TMEM163 is more closely related to ZnT9 in comparison to other ZnT proteins. Recall that TMEM163 has been reported to bind zinc, nickel, and copper [58], which suggests that this protein could potentially transport all three metals. A dual or promiscuous role in transporting metal ions should not disqualify TMEM163 to be included as a member of the ZnT family, especially that ZnT10 is now recognized as a manganese efflux transporter as well. Regardless of the controversy behind the function of ZnT9 or ZnT10, current empirical and theoretical data indicate that TMEM163 should be considered for re-classification as a new CDF family member under the mammalian SLC30 family, designated as ZnT11 and encoded by the *SLC30A11* gene [32]. Overall, TMEM163 and ZnT9 appear to be atypical members of the mammalian CDF protein family; however, more experimental evidence on function and more in-depth evolutionary analysis of the domains or sequence motifs responsible for zinc transport may eventually establish the true membership of TMEM163 and ZnT9 within the ZnT protein family. It would also be fascinating to examine whether TMEM163 serves to transport nickel or copper out of cells.

### 3.2. Protein Interactome

TMEM163 was shown to interact and partially co-localize with TRPML1, a non-selective cation channel found within lysosomes [60]. The two proteins share a similarly predicted six TMD and are both expressed in the human brain [60,65]. It is important to note that loss-of-function mutations or deletions within TRPML1 are responsible for MLIV, a rare developmental disorder and neurodegenerative disease [66]. Intriguingly, the functional relevance of TMEM163-TRPML1 interaction suggests that both proteins affect zinc homeostasis in cells [60]. This is particularly pertinent in the context of disease due to the previous observation by our laboratory that zinc accumulates in lysosomes and is elevated in MLIV patient fibroblasts [60]. Isotope analysis also revealed marked increase of zinc in post-mortem brain tissues of MLIV mouse model [67]. Fluorescence microscopy following knock down of TRPML1 in cultured human cells also showed high lysosomal zinc levels [67,68], but spectrofluorometric analysis did not necessarily reveal a significant increase of zinc concentration in cells with reduced TRPML1 expression [60]. Notwithstanding, the subcellular localization of TMEM163, its zinc transport function, and its interaction with TRPML1 all suggest that it may play a role in MLIV pathology.

There is recent evidence showing that TMEM163 has a vital role in the expression and function of P2X receptors [69]. P2X receptors are ATP-gated ion channels that serve to transmit pain signaling, and thus are a prime drug target for pain relief [70]. In their paper, Salm and colleagues (2020) revealed that TMEM163 interacts with and modulates the activity of P2X3 and P2X4 receptors [69]. Using a heterologous cell expression system, it was found that TMEM163 was able to decrease the decay of ATP-evoked currents of P2X3 receptors, thereby increasing the receptor’s activity and influencing its expression levels [69]. Similarly, ATP-evoked currents of P2X4 receptors were found to be increased upon co-expression of TMEM163 in oocytes [69]. Such an observation that TMEM163 was able to influence the function and expression of the P2X3R protein could further solidify the newly described phylogenetic relationship between ZnT9 and TMEM163 proteins [32]. This is because ZnT9 has been demonstrated also to influence and regulate specific transcriptional targets [71]. It would be remarkable to prove the hypothesis that ZnT9 and TMEM163 share a similar function in cells, which could directly impact human health.

## 4. Genome-Wide Association Study (GWAS) Implicating TMEM163 in Human Diseases

A growing number of GWAS reports has recently implicated TMEM163 as potentially causative factor in several human disorders. We summarize below the findings that link TMEM163 in PD and DM.

### 4.1. Diabetes Mellitus

One GWAS had a relatively fair number of Indian subjects with over 12,000 people in total. The study found significant association of two single nucleotide polymorphisms (SNPs) within the *TMEM163* gene with T2D: rs998451 and rs6723108. Specifically, it was hypothesized that TMEM163 may influence the secretion of insulin, due to the two SNPs being associated with lower fasting plasma insulin levels in the subjects [72]. Interestingly, a replication study using a population of 1209 people from Northwest India showed no significant association for the two SNPs rs998451 and rs6723108 with T2D [73]. However, as the authors state, India has many different ethnic groups, which makes GWAS data on T2D difficult to generalize for the whole population [73]. With this in mind, researchers in future GWAS on T2D must take into account ethnicity, especially in population with high ethnic diversity. More recently, however, a follow-up study done by another group revealed that one of the TMEM163 SNPs may indeed be linked to T2D [61]. First, the group showed that knock down of mouse Tmem163 in MIN6 cells produced elevated intracellular zinc levels [61], which is consistent with our laboratory’s earlier findings after knocking down TMEM163 in human cells [60]. The group also found that reduction of mouse Tmem163 expression in MIN6 cells resulted in decreased insulin secretion and differential expression of genes involved in glucose metabolism [61]. To further support their hypothesis that TMEM163 plays a role in T2D, the authors showed that the relative TMEM163 mRNA expression levels in human pancreatic tissue were higher compared to other tissues. [61]; however, as mentioned earlier, the apparent difference in relative levels is due to the housekeeping gene used to compare relative quantification [60]. Nevertheless, the authors reported that a novel variant of TMEM163, believed to cause a partial loss of function, was found in 33% of study participants with T2D [61]. This TMEM163 variant was found to be associated with a high glycemic index and that fasting plasma insulin level is decreased among the participants (24 patients with T2D and 24 control subjects); however, the association was not found to be significant upon further analysis [61]. As we previously mentioned, ZnT8 is also implicated in DM. Since ZnT8 and TMEM163 are expressed in pancreatic beta cells, it is thus quite conceivable that both proteins may play a redundant role in pancreatic zinc homeostasis and stabilization of insulin granules. Further studies to determine whether both ZnT8 and TMEM163 influence glucose and insulin metabolism are warranted in light of these recent observations.

Studies on gestational diabetes mellitus (GDM) of Han Chinese population reported no association with the TMEM163 SNP rs998451 [74]. The study had a sample size of 367 control pregnant women and 334 pregnant women with GDM [74]. In contrast, GWAS of Mongolian Chinese population found an association between the TMEM163 SNP rs6723108 and T2D. The study consisted of 497 subjects with T2D and 469 control subjects, which is a relatively small sample size for a GWAS report. Of interest is the fact that nearly every participant of the study has the TMEM163 SNP rs6723108 variant. However, the reason and consequences of this risk allele being at relatively high frequency remain unknown [75].

### 4.2. Parkinson’s Disease

Many of the research relating TMEM163 and PD involved GWAS and meta-analysis [76,77,78,79]. A meta-analysis consisting of around 14,000 patients, and just under 100,000 controls showed that the SNP rs6430538 (near the *ACMSD* and *TMEM163* genes) showed genome-wide significance with PD among Caucasians or European ancestry [79]. With such a large sample size, this finding strongly implicates TMEM163 in PD, but most studies that have been done focused on ACMSD [80]. Thirtamara-Rajamani and colleagues (2017) proposed that this particular SNP may be able to affect the promoters of both *ACMSD* and *TMEM163* genes, linking them to PD [80]. It would be valuable endeavor to examine whether the SNP associated with TMEM163 impacts PD etiology by performing functional studies in cells or model organisms.

A very recent report using 743 non-consanguineous Chinese patients with early-onset PD have identified multiple novel TMEM163 variants [77]. Noteworthy, the authors demonstrated direct association with PD and one variant in particular, c.98C>G (p.33P>R), was found to be significantly associated with early-onset PD [77]. The P33R amino acid substitution is located within the predicted N-terminus region of TMEM163. Although there were some limitations in the study, such as smaller sample size, lack of age- and sex-matched controls, as well as use of data only from East Asians (gnomAD v2.1.1), functional studies are warranted on this specific variant, including two other variants that were marginally associated with early-onset PD. Although TMEM163 has not been officially classified as a ZnT family member, it is worth noting that ZnT10 has been also linked to PD and dystonia [50,51,52,53]. Missense, nonsense, and nucleotide deletion within the *SLC30A10* gene produce abnormal manganese accumulation in cells that express the mutant or variant isoforms [81]. Manganese overload is invariably cytotoxic leading to PD or dystonia. In the case of association between TMEM163 and PD, the potential loss-of-function (LOF) or gain-of-function (GOF) phenotype conferred by TMEM163 variants associated with early onset PD may be attributed to imbalance of zinc, nickel or copper. While abnormal levels of zinc, copper, and rarely nickel, have been linked to various neurodegenerative diseases [82], it is unclear how protein variants of TMEM163 may contribute to PD at this point. As a future work to dissect the role of TMEM163 protein variants associated with early onset PD [77], performing functional assays to test for ionic efflux activity could show whether the variants have a LOF or GOF phenotype. Similarly, studying the subcellular localization of these TMEM163 protein variants to find out if they are mis-localized would be instructive for linking the cellular phenotype to PD etiology.

A GWAS with a Southern Spanish population that consisted of 240 patients with PD and 192 control subjects failed to find genome-wide significance with the rs6430538 variant and PD [83]. Since the sample size was not large and that some association was only found using an uncorrected *p*-value, the authors believe that having larger sample size would produce genome-wide significance with this SNP. In this vein, a replication study of another Southern Spanish population was done with a relatively larger number of people (750 patients with PD and around 1,100 control subjects) by a different set of researchers [84]. The new population’s data did demonstrate significant association between the SNP rs6430538 and PD. However, when this study performed a meta-analysis which combined the data from the previously mentioned Southern Spanish population, no genome-wide significance was found. Once again, significance between the rs6430538 locus and PD is observed when analyzed using an uncorrected *p*-value [84]. While the sample size grew larger with respect to the first study, the lack of true genomic significance in their meta-analysis leaves the true significance of this SNP, at least in this population, ostensibly unknown.

Two independent GWAS with East Asian populations attempted to determine if the SNP rs6430538 had any association in people with the aforementioned ethnic background. The first study used Taiwanese population, which had around 600 patients with PD and 600 control subjects, but found no significant association between the SNP and PD. They mentioned the importance of how the allelic frequency in their population greatly differed in some cases with Caucasian population [76]. Nevertheless, even though this SNP may not be significant for people of East Asian origin, it does not rule out possible association for others of different genetic backgrounds [76]. The second GWAS used Han Chinese population numbering around 1000 patients with sporadic PD and around 1000 control subjects [85]. Unfortunately, no association was found in this population but the authors urged that further studies need to be carried out for East Asian populations to decisively prove their results [85].

Overall, observations from GWAS using different populations may show conflicting results, which demonstrates how variable populations are and how specific SNPs may only confer an effect that is unique to certain population. It is crucial to point out how some studies such as those reported by Tan et al., (2019) [74] and Bai et al., (2015) [75] need to be replicated due to the low sample size used in their studies, and thus, future GWAS should aim to have larger populations. Additionally, a diverse set of populations should be included in the analysis of GWAS. Ethnic groups should also be taken into account due to the fact that certain parts of the world are more ethnically diverse, which could give inaccurate data if not carefully considered.

## 5. Conclusions

Over the past decade, the elucidation of TMEM163 has been slow, but progressive. Although the current observation suggesting that TMEM163 should be re-classified as ZnT11 is becoming recognized, more experimental research must be carried out to prove or disprove this proposal. Specifically, determination of more interaction partners, especially with ZnTs, would bolster this hypothesis due to the heterodimeric nature of certain SLC30 family members. Moreover, the functional importance of the TMEM163 SNPs must be further investigated because the current literature remains highly scarce. Finally, the use of larger populations, with careful consideration of the ethnic diversity within the population, must be done in all GWAS projects, in order to resolve controversial issues presented in this review. In conclusion, as mounting evidence of TMEM163’s role in the etiology of diseases comes to light, the knowledge we gain on cellular zinc homeostasis through the study of its protein transporters will remain vital to our understanding of human health and physiology.

## Figures and Tables

**Table 1:** Multiple sequence analysis of TMEM163 and ZnT protein family using Clustal Omega (MegAlign Pro, Lasergene v17.1). Percent identity and similarity are shown by the upper right values (bold text) and lower left values (regular text), respectively. The identity percentage signifies the number of amino acids that exactly matches sequences between two proteins, while similarity percentage represents the likelihood that the two proteins have evolved from a common ancestral protein. MegAlign Pro calculated the numerical values with the following parameters: distance metric = Uncorrected Pairwise; Gap treatment = Pairwise gap removal; Residues considered = 163–460.

	*TMEM163*	*ZnT1*	*ZnT2*	*ZnT3*	*ZnT4*	*ZnT5*	*ZnT6*	*ZnT7*	*ZnT8*	*ZnT9*	*ZnT10*
*TMEM163*	0	**17.6**	**10.3**	**11.5**	**11.2**	**9.5**	**11.7**	**14.7**	**10.8**	**24.2**	**18.9**
*ZnT1*	36.5	0	**30.5**	**27.1**	**25.8**	**25.6**	**16.1**	**26.5**	**27.3**	**14.5**	**38.9**
*ZnT2*	35.3	50.8	0	**53.7**	**38.4**	**22.6**	**18.4**	**22.5**	**50.6**	**14.9**	**24.8**
*ZnT3*	28.6	45.4	67.5	0	**35.1**	**20.5**	**20.2**	**22.3**	**44.5**	**13.4**	**22.7**
*ZnT4*	29.4	49.4	60.8	57.4	0	**22.6**	**18.5**	**22.4**	**39.2**	**15.4**	**25.2**
*ZnT5*	32.1	48.8	45.0	42.5	44.2	0	**24.3**	**43.6**	**21.6**	**16.0**	**25.9**
*ZnT6*	29.4	34.3	38.1	37.1	39.7	47.0	0	**24.6**	**18.2**	**14.5**	**16.7**
*ZnT7*	36.8	48.8	46.4	42.6	47.8	63.0	44.5	0	**21.8**	**15.4**	**27.8**
*ZnT8*	30.8	52.3	69.1	64.3	61.0	44.1	37.9	42.9	0	**14.9**	**25.7**
*ZnT9*	46.1	36.6	36.1	31.4	34.0	34.4	30.1	35.2	33.4	0	**17.3**
*ZnT10*	36.0	54.1	47.6	44.5	48.8	43.7	38.0	45.8	50.0	37.3	0

## Data Availability

Protein sequences used for MSA and phylogeny data analyses are available upon request.
